# Supplementary material for: Effect of Facial Skin Temperature on the Perception of Anxiety: A Pilot Study
Source: Healthcare (Basel). 2020 Jul 9;8(3):206. doi: 10.3390/healthcare8030206 (PMC7551020; doi:10.3390/healthcare8030206)
Supplement: Supplementary file 1 [file healthcare-08-00206-s001.pdf]

**Table S1.** Mean scores between groups for the CPR quality parameters and knowledge, stress and anxiety questionnaires.

| CPR Parameters/ STAI Scores | Study Moment | Uudergraduate (BS) | *p* Value (Pre-/Post-test | Postgraduate (MS) | p Value (Pre-/Post-test | *p* Value |
|---|---|---|---|---|---|---|
| | | Score Mean (SD) | | Score Mean (SD) | | |
| Knowledge questionnaire (%) | Success Rate (%) | 70.83 (19.50) | | 82.24 (10.47) | | 0.026 $^{*,a}$ |
| | Correct answers | 5.67 (1.56) | | 6.58(.84) | | 0.037 $^{*,b}$ |
| CPR global score (%) | | 68.24 (17.78) | | 80.74 (16.33) | | 0.027 $^{*,b}$ |
| Number of compressions | | 163.81 (15.98) | | 156.11 (17.88) | | 0.198 $^{c}$ |
| Compressions proper depth (%) | | 90.81 (23.09) | | 98.53 (3.86) | | 0.290 $^{c}$ |
| Compressions proper expansion (%) | | 77.52 (32) | | 74.05 (30.98) | | 0.430 $^{c}$ |
| Compressions rate adequate (%) | | 47.10 (38.73) | | 53.26 (38.57) | | 0.559 $^{c}$ |
| Mean Compressions rate 1 minute | | 122.48 (11.64) | | 110.84 (23.32) | | 0.002 $^{*,c}$ |
| Time spent without compressions interruption (seconds) | | 68.81 (3.61) | | 72.42 (4.51) | | 0.008 $^{*,a}$ |
| Ventilations global score (%) | | 31.43 (39.83) | | 55.42 (42.76) | | 0.085 $^{c}$ |
| Number of ventilations | | 3.38 (4.48) | | 5.63 (4.39) | | 0.209 $^{c}$ |
| Ventilations adequate volume (%) | | 43.86 (49.391) | | 59.74 (46.21) | | 0.324 $^{c}$ |
| Ventilations excessive volume (%) | | 3.76 (15.303) | | 8.74±20.83 | | 0.295 $^{c}$ |
| Stress visual analogue scale score (VAS) | Pre-test | 3.24 (1.97) | 0.874 $^{d}$ | 2.74 (1.82) | 0.635 $^{d}$ | 0.287 $^{c}$ |
| | Post-test | 3.40 (2.26) | | 3.03 (2.59) | | 0.502 $^{c}$ |
| State-Trait anxiety inventory score (STAI) | Pre-test | 24.24 (4.74) | 0.752 $^{d}$ | 22.68 (5.07) | 0.084 $^{d}$ | 0.323 $^{a}$ |
| | Post-test | 23.90 (4.812) | | 24.95 (5.20) | | 0.514 $^{a}$ |

* $p < 0.05$; ** $p < 0.01$; $^{a}$ t-Student independent samples; $^{b}$ Chi-Squared Pearson; $^{c}$ Mann–Whitney U; $^{d}$ t-Student paired samples.

**Table S2.** Bivariate correlations between STAI scores and temperature values of the selected regions of interest before and after simulation.

| Facial Region | Point | Group | Statistic and *p* Value Title | STAI Pre-Test | STAI Post-Test |
|---|---|---|---|---|---|
| **Nose** | Average | Undergraduate (BS) | Correlation coefficient | −0.184 | −0.262 |
| | | | Significance | 0.426 | 0.251 |
| | | Postgraduate (MS) | Correlation coefficient | 0.379 | −0.080 |
| | | | Significance | 0.109 | 0.744 |
| **Forehead** | Maximum | Undergraduate (BS) | Correlation coefficient | 0.627 ** | 0.537 * |
| | | | Significance | 0.002 | 0.012 |
| | | Postgraduate (MS) | Correlation coefficient | 0.499 * | −0.051 |
| | | | Significance | 0.030 | 0.835 |
| | Average | Undergraduate (BNS) | Correlation coefficient | 0.331 | 0.553 * |
| | | | Significance | 0.143 | 0.009 |
| | | Postgraduate (MS) | Correlation coefficient | 0.440 | 0.068 |
| | | | Significance | 0.060 | 0.782 |
| | Minimum | Undergraduate (BS) | Correlation coefficient | −0.157 | 0.305 |
| | | | Significance | 0.495 | 0.179 |
| | | Postgraduate (MS) | Correlation coefficient | 0.086 | 0.141 |
| | | | Significance | 0.727 | 0.566 |
| **Periorbital** | Maximum | Undergraduate (BS) | Correlation coefficient | 0.324 | 0.323 |
| | | | Significance | 0.152 | 0.153 |
| | | Postgraduate (MS) | Correlation coefficient | 0.473 * | 0.092 |
| | | | Significance | 0.042 | 0.708 |
| | Average | Undergraduate (BNS) | Correlation coefficient | 0.145 | 0.391 |
| | | | Significance | 0.529 | 0.080 |
| | | Postgraduate (MS) | Correlation coefficient | 0.509 * | −0.059 |
| | | | Significance | 0.026 | 0.812 |
| | Minimum | Undergraduate (BNS) | Correlation coefficient | −0.178 | −0.132 |
| | | | Significance | 0.440 | 0.568 |
| | | Postgraduate (MS) | Correlation coefficient | 0.332 | −0.249 |
| | | | Significance | 0.166 | 0.304 |
| **Maxillary** | Maximum | Undergraduate (BS) | Correlation coefficient | 0.120 | 0.035 |
| | | | Significance | 0.604 | 0.880 |
| | | Postgraduate (MS) | Correlation coefficient | 0.537 * | 0.029 |

| | | | | | |
|---|---|---|---|---|---|
| | | | Significance | 0.018 | 0.906 |
| | Average | Undergraduate (BS) | Correlation coefficient | 0.133 | 0.020 |
| | | | Significance | 0.566 | 0.932 |
| | | Postgraduate (MS) | Correlation coefficient | 0.534 * | 0.049 |
| | | | Significance | 0.019 | 0.843 |
| | Minimum | Undergraduate (BS) | Correlation coefficient | 0.013 | −0.139 |
| | | | Significance | 0.957 | 0.546 |
| | | Postgraduate (MS) | Correlation coefficient | 0.388 | 0.057 |
| | | | Significance | 0.101 | 0.816 |
| **Neck / Upper chest** | Maximum | Undergraduate (BN) | Correlation coefficient | 0.381 | 0.332 |
| | | | Significance | 0.089 | 0.142 |
| | | Postgraduate (MS) | Correlation coefficient | 0.115 | −0.135 |
| | | | Significance | 0.640 | 0.581 |
| | Average | Undergraduate (BS) | Correlation coefficient | 0.381 | 0.559 ** |
| | | | Significance | 0.089 | 0.008 |
| | | Postgraduate (MS) | Correlation coefficient | 0.142 | 0.120 |
| | | | Significance | 0.563 | 0.624 |
| | Minimum | Undergraduate (BS) | Correlation coefficient | 0.104 | −0.036 |
| | | | Significance | 0.652 | 0.877 |
| | | Postgraduate (MS) | Correlation coefficient | 0.230 | 0.269 |
| | | | Significance | 0.344 | 0.265 |

* $p < 0.05$; ** $p < 0.01$; Pearson correlation coefficients.

**Table S3.** Bivariate correlations between CPR quality parameters and temperature values of the selected regions of interest for the Undergraduate (BS) Group.

| Temperature Difference | Statistic and *p* Value | CPR Global Score [a] | Number of Compressions [b] | Compressions Proper Depth [b] | Compressions Proper Expansion [b] | Compressions Rate Adequate [b] | Mean Compressions Rate [b] | Time without Interruption [a] | Ventilations Global Score [b] | Number of Ventilations [b] | Ventilations Adequate Volume [b] | Ventilations Excessive Volume [b] |
|---|---|---|---|---|---|---|---|---|---|---|---|---|
| **Nose** | Correlation coefficient | 0.292 | 0.280 | 0.223 | −0.396 | −0.207 | 0.307 | −0.054 | 0.121 | 0.188 | 0.056 | 0.285 |
| | Significance | 0.199 | 0.218 | 0.332 | 0.075 | 0.367 | 0.175 | 0.816 | 0.602 | 0.416 | 0.811 | 0.211 |
| **Forehead maximum** | Correlation coefficient | 0.377 | −0.373 | 0.027 | 0.239 | 0.295 | −0.264 | −0.199 | 0.198 | 0.242 | 0.355 | −0.057 |
| | Significance | 0.092 | 0.096 | 0.908 | 0.296 | 0.193 | 0.247 | 0.388 | 0.390 | 0.291 | 0.115 | 0.805 |
| **Forehead average** | Correlation coefficient | 0.379 | −0.406 | 0.083 | 0.238 | 0.327 | −0.310 | −0.111 | 0.081 | 0.115 | 0.311 | −0.204 |
| | Significance | 0.090 | 0.068 | 0.721 | 0.299 | 0.148 | 0.171 | 0.633 | 0.728 | 0.619 | 0.170 | 0.375 |
| **Forehead minimum** | Correlation coefficient | 0.108 | −0.322 | −0.111 | −0.073 | 0.445 * | −0.395 | −0.170 | 0.175 | 0.247 | 0.159 | 0.329 |
| | Significance | 0.643 | 0.155 | 0.632 | 0.752 | 0.043 | 0.076 | 0.463 | 0.447 | 0.280 | 0.492 | 0.146 |
| **Periorbital maximum** | Correlation coefficient | 0.272 | −0.514 * | −0.238 | 0.215 | 0.331 | −0.407 | −0.304 | 0.344 | 0.377 | 0.379 | 0.166 |
| | Significance | 0.232 | 0.017 | 0.298 | 0.350 | 0.153 | 0.067 | 0.181 | 0.127 | 0.092 | 0.090 | 0.472 |
| **Periorbital average** | Correlation coefficient | 0.268 | −0.360 | 0.178 | 0.009 | 0.332 | −0.324 | −0.160 | 0.124 | 0.179 | 0.216 | 0.060 |
| | Significance | 0.241 | 0.109 | 0.440 | 0.971 | 0.141 | 0.152 | 0.489 | 0.591 | 0.437 | 0.348 | 0.796 |
| **Periorbital minimum** | Correlation coefficient | 0.420 | 0.015 | 0.097 | −0.014 | 0.024 | −0.063 | 0.062 | 0.201 | 0.274 | 0.263 | 0.222 |
| | Significance | 0.058 | 0.948 | 0.675 | 0.950 | 0.917 | 0.787 | 0.788 | 0.383 | 0.229 | 0.249 | 0.334 |
| **Maxillary maximum** | Correlation coefficient | 0.406 | −0.237 | 0.021 | 0.112 | 0.316 | −0.234 | 0.027 | 0.138 | 0.201 | 0.222 | 0.128 |
| | Significance | 0.067 | 0.300 | 0.928 | 0.630 | 0.164 | 0.307 | 0.909 | 0.552 | 0.382 | 0.333 | 0.581 |
| **Maxillary average** | Correlation coefficient | 0.393 | −0.059 | 0.124 | −0.013 | 0.145 | −0.065 | −0.018 | 0.066 | 0.117 | 0.155 | 0.042 |
| | Significance | 0.078 | 0.801 | 0.592 | 0.955 | 0.531 | 0.778 | 0.939 | 0.776 | 0.613 | 0.503 | 0.856 |
| **Maxillary minimum** | Correlation coefficient | 0.351 | 0.435 * | 0.263 | −0.328 | −0.277 | 0.300 | 0.215 | 0.069 | 0.128 | 0.033 | 0.279 |
| | Significance | 0.119 | 0.049 | 0.249 | 0.146 | 0.223 | 0.186 | 0.350 | 0.767 | 0.580 | 0.886 | 0.220 |
| **Neck maximum** | Correlation coefficient | 0.432 | −0.313 | −0.004 | 0.182 | 0.342 | −0.221 | −0.399 | −0.012 | 0.017 | 0.044 | −0.111 |
| | Significance | 0.050 | 0.167 | 0.988 | 0.429 | 0.130 | 0.336 | 0.073 | 0.959 | 0.943 | 0.850 | 0.632 |
| **Neck average** | Correlation coefficient | 0.401 | −0.212 | 0.170 | 0.124 | 0.354 | −0.228 | −0.227 | 0.164 | 0.234 | 0.179 | 0.101 |
| | Significance | 0.072 | 0.356 | 0.461 | 0.592 | 0.115 | 0.321 | 0.322 | 0.477 | 0.308 | 0.437 | 0.664 |
| **Neck minimum** | Correlation coefficient | 0.091 | 0.012 | 0.111 | −0.287 | 0.147 | −0.146 | 0.025 | 0.196 | 0.193 | 0.059 | 0.199 |

| | | | | | | | | | | | | |
|---|---|---|---|---|---|---|---|---|---|---|---|---|
| | Significance | 0.694 | 0.959 | 0.633 | 0.207 | 0.524 | 0.529 | 0.915 | 0.395 | 0.401 | 0.799 | 0.388 |

[a] Pearson's R correlation coefficient; [b] Spearman's Rho correlation coefficient; * $p < 0.05$

**Table S4.** Bivariate correlations between CPR quality parameters and temperature values of the selected regions of interest for the Postgraduate (MS) Group.

| Temperature Difference | Statistic and p Value | CPR Global Score [a] | Number of Compressions [b] | Compressions Proper Depth [b] | Compressions Proper Expansion [b] | Compressions Rate Adequate [b] | Mean Compressions Rate [b] | Time without Compressions [a] | Ventilations Global Score [b] | Number of Ventilations [b] | Ventilations Adequate Volume [b] | Ventilations Excessive Volume [b] |
|---|---|---|---|---|---|---|---|---|---|---|---|---|
| **Nose** | Correlation coefficient | 0.203 | −0.524 * | 0.469 * | −0.027 | −0.088 | −0.278 | −0.075 | 0.294 | 0.126 | −0.029 | 0.247 |
| | Significance | 0.405 | 0.021 | 0.043 | 0.913 | 0.720 | 0.249 | 0.761 | 0.222 | 0.606 | 0.907 | 0.309 |
| **Forehead maximum** | Correlation coefficient | 0.105 | −0.341 | 0.233 | −0.067 | −0.210 | −0.331 | 0.125 | 0.257 | −0.021 | 0.052 | −0.136 |
| | Significance | 0.668 | 0.153 | 0.336 | 0.786 | 0.388 | 0.167 | 0.609 | 0.289 | 0.933 | 0.833 | 0.578 |
| **Forehead average** | Correlation coefficient | 0.145 | −0.310 | 0.094 | 0.037 | −0.041 | −0.180 | 0.011 | 0.446 | 0.122 | 0.096 | −0.129 |
| | Significance | 0.553 | 0.196 | 0.703 | 0.880 | 0.866 | 0.460 | 0.965 | 0.055 | 0.618 | 0.697 | 0.597 |
| **Forehead minimum** | Correlation coefficient | −0.083 | 00.069 | 0.292 | 0.394 | −0.384 | −0.109 | 0.183 | −0.050 | −0.072 | −0.089 | −0.327 |
| | Significance | 0.736 | 0.780 | 0.225 | 0.095 | 0.104 | 0.657 | 0.454 | 0.839 | 0.770 | 0.718 | 0.172 |
| **Periorbital maximum** | Correlation coefficient | 0.019 | −0.248 | 0.219 | −0.052 | −0.132 | −0.299 | 0.279 | 0.204 | 0.068 | −0.055 | 0.005 |
| | Significance | 0.939 | 0.305 | 0.367 | 0.831 | 0.591 | 0.213 | 0.248 | 0.402 | 0.784 | 0.824 | 0.984 |
| **Periorbital average** | Correlation coefficient | 0.159 | −0.291 | 0.252 | 0.052 | −0.022 | −0.233 | 0.085 | 0.431 | 0.248 | 0.088 | 0.156 |
| | Significance | 0.516 | 0.226 | 0.298 | 0.834 | 0.930 | 0.338 | 0.730 | 0.065 | 0.305 | 0.719 | 0.522 |
| **Periorbital minimum** | Correlation coefficient | 0.132 | −0.182 | 00.129 | −0.329 | 0.100 | 0.126 | −0.284 | 0.301 | 0.188 | 0.010 | 0.274 |
| | Significance | 0.589 | 0.455 | 0.599 | 0.170 | 0.682 | 0.608 | 0.238 | 0.210 | 0.442 | 0.966 | 0.257 |
| **Maxillary maximum** | Correlation coefficient | 0.031 | −0.282 | −0.078 | 0.050 | 0.179 | -0.270 | 0.103 | 0.325 | 0.131 | 0.069 | 0.010 |
| | Significance | 0.898 | 0.241 | 0.752 | 0.839 | 0.463 | 0.264 | 0.675 | 0.174 | 0.594 | 0.780 | 0.968 |
| **Maxillary average** | Correlation coefficient | 0.025 | −0.367 | 0.162 | −0.100 | 0.026 | −0.281 | 0.052 | 0.178 | 0.033 | 0.003 | 0.036 |
| | Significance | 0.918 | 0.123 | 0.507 | 0.683 | 0.917 | 0.243 | 0.833 | 0.467 | 0.892 | 0.991 | 0.885 |
| **Maxillary minimum** | Correlation coefficient | −0.001 | −0.677 ** | 0.111 | −0.179 | 0.038 | −0.423 | −0.173 | −0.052 | −0.145 | −0.232 | 0.277 |
| | Significance | 0.997 | 0.001 | 0.650 | 0.464 | 0.878 | 0.071 | 0.480 | 0.833 | 0.553 | 0.340 | 0.251 |
| **Neck maximum** | Correlation coefficient | −0.009 | 0.137 | 0.101 | −0.248 | −0.242 | 0.054 | 0.217 | −0.027 | 0.307 | −0.118 | 0.400 |
| | Significance | 0.972 | 0.577 | 0.680 | 0.305 | 0.319 | 0.826 | 0.371 | 0.912 | 0.201 | 0.631 | 0.090 |
| **Neck average** | Correlation coefficient | 0.065 | −0.146 | 0.186 | −0.180 | −0.244 | −0.075 | 0.048 | 0.062 | 0.261 | −0.075 | 0.423 |
| | Significance | 0.793 | 0.551 | 0.445 | 0.460 | 0.314 | 0.761 | 0.845 | 0.800 | 0.281 | 0.759 | 0.071 |

| | | | | | | | | | | | | |
|---|---|---|---|---|---|---|---|---|---|---|---|---|
| **Neck minimum** | Correlation coefficient | −0.322 | 0.324 | −0.052 | −0.399 | 0.022 | −0.537 * | −0.183 | −0.265 | −0.343 | −0.042 | −0.453 |
| | Significance | 0.178 | 0.176 | 0.832 | 0.090 | 0.929 | 0.018 | 0.452 | 0.273 | 0.150 | 0.863 | 0.052 |

[a] Pearson's R correlation coefficient; [b] Spearman's Rho correlation coefficient; * $p < 0.05$; ** $p < 0.01$.
